# Pathological brain detection in MRI scanning by wavelet packet Tsallis entropy and fuzzy support vector machine

**DOI:** 10.1186/s40064-015-1523-4

**Published:** 2015-11-24

**Authors:** Yu-Dong Zhang, Shui-Hua Wang, Xiao-Jun Yang, Zheng-Chao Dong, Ge Liu, Preetha Phillips, Ti-Fei Yuan

**Affiliations:** 1 School of Computer Science and Technology, Nanjing Normal University, Nanjing, Jiangsu 210023 China; 2 Jiangsu Key Laboratory of 3D Printing Equipment and Manufacturing, Nanjing, Jiangsu 210042 China; 3 Department of Mathematics and Mechanics, China University of Mining and Technology, Xuzhou, Jiangsu 221008 China; 4 Translational Imaging Division and MRI Unit, Columbia University and New York State Psychiatric Institute, New York, NY 10032 USA; 5 Department of Psychiatry, College of Physicians and Surgeons, Columbia University, New York, NY 10032 USA; 6 School of Natural Sciences and Mathematics, Shepherd University, Shepherdstown, WV 25443 USA; 7 School of Psychology, Nanjing Normal University, Nanjing, Jiangsu 210008 China

**Keywords:** Pathological brain detection (PBD), Tsallis entropy, Magnetic resonance imaging, Computer-aided diagnosis, Discrete wavelet packet transform, Fuzzy support vector machine, Pattern recognition

## Abstract

An computer-aided diagnosis system of pathological brain detection (PBD) is important for help physicians interpret and analyze medical images. We proposed a novel automatic PBD to distinguish pathological brains from healthy brains in magnetic resonance imaging scanning in this paper. The proposed method simplified the PBD problem to a binary classification task. We extracted the wavelet packet Tsallis entropy (WPTE) from each brain image. The WPTE is the Tsallis entropy of the coefficients of the discrete wavelet packet transform. The, the features were submitted to the fuzzy support vector machine (FSVM). We tested the proposed diagnosis method on 3 benchmark datasets with different sizes. A ten runs of K-fold stratified cross validation was carried out. The results demonstrated that the proposed WPTE + FSVM method excelled 17 state-of-the-art methods w.r.t. classification accuracy. The WPTE is superior to discrete wavelet transform. The Tsallis entropy performs better than Shannon entropy. The FSVM excels standard SVM. In closing, the proposed method “WPTE + FSVM” is effective in PBD.

## Background

Pathological brain detection (PBD) was of essential importance. It can help physicians make decisions, and to avoid wrong judgements on subjects. Magnetic resonance imaging (MRI) features in high-resolution on soft tissues in the subjects’ brains, generating a mass dataset (Zhang et al. 2015a). At present, there are numerous works on using brain MR images for solving PBD problems (Goh et al. 2014; Yu et al. 2015b).

Recent computer-aided diagnosis (CAD) systems of PBD consisted of two types (LaViolette et al. 2014): to detect pathological from healthy brains, and to differentiate severity degrees. In this study, we research on the former one. A type of promising approach is to use discrete wavelet transform (DWT) that presents the solutions of simultaneous analysis in domains of both time and frequency (Lee et al. 2013; Dong et al. 2014; Zhang et al. 2015c; Yu et al. 2015c). DWT and its variants achieved good results; however, DWT are translation-variant, hence, the coefficients behaved unpredictably if the input signal is translated slightly. In PBD problem, the subject’s head usually have slightly move during the scan, which will cause the translation of MR images.

Another problems is the classifier. Current scholars tend to use either artificial neural network (ANN) or support vector machine (SVM). Nevertheless, both of them are sensitive to outliers and noises. That means, if the training set contains noises or outliers, the classifier will still treat it as important as normal data.

We suggested three improvements with the aim of solving above problems. First, we employed the discrete version of wavelet packet transform (WPT), which is an extension of standard discrete wavelet transform (DWT). Second, we introduced Tsallis entropy (TE), to replace with Shannon entropy (SE). (iii) We introduced the fuzzy support vector machine (FSVM) that combines the SVM with fuzzy logic approach (Ashkezari et al. 2013) and has the advantage of reducing the effect from outliers and noises.

The structure of the rest is organized as follows. "State-of-the-art" presents the state-of-the-art. "Materials" introduces the materials used in this study.  “Feature extraction" discusses the features. "Classifier" gives the classifier.  "Implementation and experiments" shows the implementation of the whole method, and designs the experiments. "Results and dicussion" contains the results and discussions. "Conclusion and future research" offers conclusion and future research. We explain the nomenclatures in Abbreviations at the end of the paper.

## State-of-the-art

Chaplot et al. (2006) was the first to solve PBD problem. They used the approximation coefficients from DWT, and utilized the support vector machine (SVM) and self-organizing map (SOM). El-Dahshan et al. (2010) extracted all coefficients of all subbands of a three-level discrete wavelet transform (DWT). Then, they reduced the size of features by principal component analysis (PCA). Finally, two classifiers, K-nearest neighbors (KNN) and feed-forward back-propagation ANN (FP-ANN), were employed. Wu and Wang (2011) followed EI-Dahshan’s method, but suggest to use a feed-forward neural network (FNN) as the classifier, which was trained by scaled chaotic artificial bee colony (SCABC). Dong et al. (2011) proposed to employed scaled conjugate gradient (SCG) method to take place of SCABC. Zhang and Wu (2012) suggested to utilize kernel support vector machine (KSVM). 3 kernels were provided such as homogeneous and inhomogeneous polynomial, and radial basis function (RBF). Das et al. (2013) developed a novel method as Ripplet transform (RT) + principal component analysis (PCA) + least square support vector machine (LS-SVM). Their five-fold cross validation results showed promising classification accuracies. Saritha et al. (2013) proposed a novel feature of wavelet-entropy (WE), and employed spider-web plots (SWP) to further reduce features. Afterwards, they used the probabilistic neural network (PNN). Yu et al. (2015d) commented on Saritha’s paper and stated that dropping the SWP can obtain the same results. Zhang et al. (2013) suggested to use particle swarm optimization to train KSVM. Padma and Sukanesh (2014) used combined wavelet statistical texture features, to segment and classify AD benign and malignant tumor slices. El-Dahshan et al. (2014) used the feedback pulse-coupled neural network for image segmentation, the DWT for features extraction, the PCA for reducing the dimensionality of the wavelet coefficients, and the FBPNN to classify inputs into normal or abnormal. Wang et al. (2014) used kernel support vector machine decision tree. Zhou et al. (2015) used wavelet-entropy as the feature space, then they employed a Naive Bayes classifier (NBC) classification method. Their results over 64 images showed that the sensitivity of the classifier was 94.50 %, the specificity 91.70 %, the overall accuracy 92.60 %. Damodharan and Raghavan (2015) combined tissue segmentation and neural network for brain tumor detection. Yang et al. (2015) selected wavelet-energy as the features, and introduced biogeography-based optimization (BBO) to train the SVM. Their method reached 97.78 % accuracy on 90 T2-weighted MR brain images. Nazir et al. (2015) suggested to use filters for the removal of noises, and extracted color moments as mean features. Finally, they achieved an overall accuracy of 91.8 %. Dong et al. (2015) suggested to use a 3D eigenbrain method to detect subjects and brain regions related to AD. The accuracy achieved 92.36 ± 0.94. Harikumar and Kumar (2015) analyzed the performance of ANN, in terms of classification of medical images, using wavelets as feature extractor. Their classification accuracy achieved 96 %. Wang et al. (2015a) suggested to use stationary wavelet transform (SWT) to replace DWT, and then they proposed a Hybridization of Particle swarm optimization and Artificial bee colony (HPA) algorithm to train the classifier. Farzan et al. (2015) used longitudinal percentage of brain volume changes (PBVC) in two-year follow up and its intermediate counterparts in early 6-month and late 18-month as features. Their experiment results obtained accuracy of 91.7 %. Munteanu et al. (2015) employed Proton Magnetic Resonance Spectroscopy (MRS) data, with the aim of detecting MCI and AD. They used a single-layer perceptron with only two spectroscopic voxel volumes obtained in the left hippocampus, with an AUROC value of 0.866. Zhang et al. (2015d) combined wavelet entropy with Hu moment invariants (HMI). The feature number is in total 14. They also used GEPSVM as the classifier.

## Materials

### Magnetic resonance brain image dataset

**Fig. 1:**
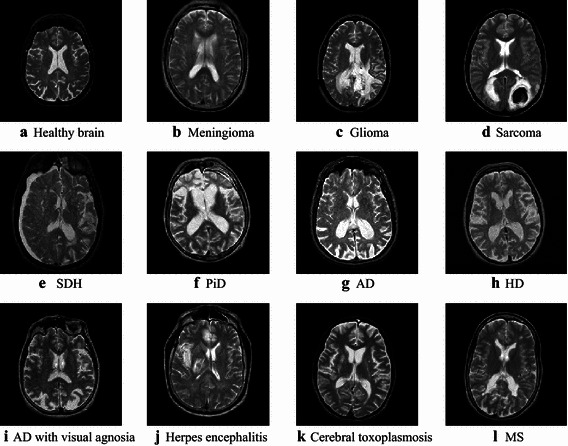
Sample of magnetic resonance brain image dataset **a** Healthy brain, **b** Meningioma, **c** Glioma, **d** Sarcoma, **e** SDH, **f** PiD, **g** AD, **h** HD, **i** AD with visual agnosia, **j** Herpes encephalitis, **k** Cerebral toxoplasmosis, **l** MS

Three benchmark magnetic resonance brain image datasets with various image numbers: D-66, D-160, and D-255, were were downloaded from the website of Harvard University. Those data contain T2-weighted images obtained along axial plane. Their sizes are all 256 × 256. Those three datasets are commonly used in PBD test. Except healthy brain images, D-66 and D-160 consisted of 7 types of brain diseases: AD, AD plus visual agnosia, glioma, meningioma, sarcoma, Huntington’s disease (HD), and Pick’s disease (PiD). D-255 introduced four other diseases as cerebral toxoplasmosis, subdural hematoma (SDH), multiple sclerosis (MS), and herpes encephalitis. Figure 1 shows samples of brain MR images.

The costs of two kinds of misclassifications are different. The cost of predicting a pathological brain to a healthy one is very serious. It will defer the necessary treatment, whereas the misprediction of a healthy brain to a pathological one can be second-checked by other techniques. Hence, we intentionally create the three imbalanced datasets, which covers more pathological brains than usual, so the PBD system is biased to detect pathological ones, with the aim of addressing this cost-sensitive task.

### Statistical setting

Cross validation (CV) is commonly used for statistical test. Stratification is embedded to CV so that each fold contains nearly the same class distributions. In this work, six-fold stratified CV (SCV) was utilized for the smallest dataset (D-66), and five-fold SCV for the other datasets (D-160 and D-255). Table 1 lists the SCV setting of all datasets.

**Table 1:** SCV setting of our datasets

Dataset	Total		Training		Validation		Fold #
	H	P	H	P	H	P	
D-66	18	48	15	40	3	8	6-
D-160	20	140	16	112	4	28	5-
D-255	35	220	28	176	7	44	5-

*P* Pathological,* H* Healthy

## Feature extraction

Co-registration was unnecessary since many publications about PBD did not use it with excellent classification results, comparative with the results that employed coregistration (Ribbens et al. 2014; Schwarz and Kasparek 2014).

### Wavelet packet transform

Compared to standard discrete wavelet transform (DWT), the wavelet packet transform (WPT) is an extension where the signal is passed through more filters than DWT. The DWT calculate each level by passing only the previous approximation coefficients to quadrature mirror filters (QMF). Nevertheless, the WPT passes all coefficients (both approximation and detail) through QMF to create a full binary tree. Therefore, more features can be generated by WPT at different levels to obtain more information. The mathematical equation of WPT is given below

1 $$S_{p}^{m,d} = \int_{ - \infty }^{\infty } {x(t)\psi_{m} (2^{ - d} t - p){\text{d}}t}$$

where *m* represents the index of channel, *p* the position parameter, *d* the decomposition level, *ψ* the wavelet function, and *S* the decomposition coefficients. 2^*d*^ sequences will be yielded at the *d* level. The decomposition equations of next level is provided as

2 $$S_{k}^{2m,d + 1} = \sum\limits_{p \in Z} {h(p - 2k)S_{p}^{m,d} }$$

3 $$S_{k}^{2m + 1,d + 1} = \sum\limits_{p \in Z} {l(p - 2k)S_{p}^{m,d} }$$

**Fig. 2:**
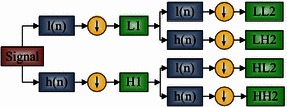
Flowchart of 2-level 1D-WPT

Suppose a *d*-level decomposition, DWT produces (3*d* + 1) coefficient sets, while the WPT produces 2^*d*^ different coefficients sets. Note that the number of coefficients of WPT is still the same of DWT, because of the downsampling process (Fig. 2).

### Shannon and Tsallis entropy

Shannon entropy (SE) is defined as a measure of uncertainty regarding the information content (IC):

4 $$E = - \sum\limits_{k = 1}^{Z} {p_{k} \log_{2} (p_{k} )}$$

here E represents the entropy, Z the total number of greylevels, k the greylevel, and pk the probability of k. Shannon entropy can merely describes scenarios with simple effective microscopic interactions and short-ranged microscopic memory (Campos 2010). Assume a physical system can be broken down into two independent subsystems X and Y, then the Shannon entropy (SE) exists the additivity property as

5 $$E(X + Y) = E(X) + E(Y)$$

Nevertheless, realistic scenarios are usually usually involved with long-time memory and long-range interactions, therefore, Tsallis (2009) proposed a generalization of SE. He termed it as Tsallis entropy (TE) with following form

6 $$E_{q} = \frac{{\sum\limits_{k = 1}^{q} {(p_{k} )^{q} } - 1}}{1 - q}$$

here *q* is a real number, representing the nonextensivity degree. For a statistical dependent system, the Tsallis entropy (TE) is defined as (Zhang and Wu 2011)

7 $$E_{q} (X + Y) = E_{q} (X) + E_{q} (Y) + (1 - q) \times E_{q} (X) \times E_{q} (Y)$$

This equation obeys the pseudo additivity rule. Further, three different entropies can be deduced and listed in Table 2, when *q* is assigned with different values (Tsallis 2011). In this study, TE was employed to extract features from 16 subbands of WPT coefficients of MR brain images.

**Table 2:** Properties of TE change with *q*

Range of *q*	Type	Property
<1	Subextensive entropy	*E*_*q*_(*A* + *B*) < *E*_*q*_(*A*) + *E*_*q*_(*B*)
=1	Standard extensive entropy (Shannon entropy)	*E*_*q*_(*A* + *B*) = *E*_*q*_(*A*) + *E*_*q*_(*B*)
>1	Superextensive entropy	*E*_*q*_(*A* + *B*) > *E*_*q*_(*A*) + *S*_*q*_(*B*)

### Wavelet packet Tsallis entropy

We employed both Shannon entropy (SE) and Tsallis entropy (TE) to extract wavelet-packet decomposition coefficients. The final extracted features were dubbed as Wavelet Packet Tsallis Entropy (WPTE), which degraded to Wavelet Packet Shannon Entropy (WPSE) when *q* equals to 1. The pseudocodes of feature extraction were listed in Table 3.

**Table 3:** Pseudocode of WPTE

Algorithm: WPTE Extraction	
Step A	Import a brain image
Step B	Implement a two-level WPT decomposition
Step C	Extract the Tsallis entropy over each coefficient set
Step D	Output the 16-element WPTE vector

## Classifier

### Support vector machine

Let us suppose there is an *N*-size training samples of *p*-dimensional vector in two classes (−1 or +1), and the goal is to create a (*p* − 1)-dimensional hyperplane. Assume the dataset takes the form of (Wang et al. 2014)

8 $$\left\{ {(x_{n} ,y_{n} )|x_{n} \in {\mathbb{R}}^{p} ,y_{n} \in \{ + 1, -1\} } \right\},n = 1,2,3,..,N$$

where *y*_*n*_ takes the value of −1 for class −1, or +1 for class +1. The *x*_*n*_ denotes a training point that is a *p*-dimensional vector (Zhang et al. 2013). The maximum-margin hyperplane that separates the two classes is the desired SVM. Considering any hyperplane is in the form of ${\mathbf{wx}} - {\mathbf{b}} = 0$, we need to select the optimal **b** and **w**, with the aim of maximizing the distance between the two parallel hyperplanes, while it can yet separate the data of the two classes.

9 $$\begin{array} {l} \mathop {\hbox{min} }\limits_{{{\mathbf{b}},{\mathbf{w}}}} \frac{1}{2}\left\| {\mathbf{w}} \right\|^{2} \hfill \\ {\text{s}} . {\text{t}} .\, { }y_{n} \left( {{\mathbf{w}}x_{n} - {\mathbf{b}}} \right) \ge 1, \,\, n = 1,2,3, \ldots ,N \hfill \\ \end{array}$$

Positive slack vector **ξ** = (*ξ*_1_, …, *ξ*_*n*_, …, *ξ*_*N*_) are utilized to measure the misclassification rate of sample *x*_*n*_ (the distance between the margin and the vectors *x*_*n*_ on the wrong side). The optimal hyperplane can be deduced by solving:

10 $$\begin{array}{*{20}l} \mathop {\hbox{min} }\limits_{{{\mathbf{w}},\xi ,{\mathbf{b}}}} \frac{1}{2}\left\| {\mathbf{w}} \right\|^{2} + Ce^{T} {\varvec{\upxi}} \hfill \\ s.t. \, \left\{ {\begin{array}{*{20}c} {y_{n} \left( {{\mathbf{w}}^{T} x_{n} - {\mathbf{b}}} \right) \ge 1 - \xi_{n} } \\ {\xi_{n} \ge 0} \\ \end{array} } \right., \,\, n = 1, \ldots ,N \hfill \\ \end{array}$$

where *C* represents the error penalty and *e* a vector of ones of *N*-dimension. Therefore, the optimization turns to a trade-off between a large margin and a small error penalty. The constraint optimization problem can be solved using “*Lagrange multiplier*” as

11 $$\mathop {\hbox{min} }\limits_{{{\mathbf{w}},\xi ,{\mathbf{b}}}} \mathop {\hbox{max} }\limits_{\alpha ,\beta } \left\{ {\frac{1}{2}\left\| {\mathbf{w}} \right\|^{2} + Ce^{T} {\varvec{\upxi}} - \sum\limits_{n = 1}^{N} {\alpha_{n} \left[ {y_{n} \left( {{\mathbf{w}}^{T} x_{n} - {\mathbf{b}}} \right) - 1 + \xi_{n} } \right] - \sum\limits_{n = 1}^{N} {\beta_{n} \xi_{n} } } } \right\}$$

The min–max problem is not easy to solve, so *dual form* technique is commonly proposed to solve it as

12 $$\begin{array}{l} \mathop {\hbox{max} }\limits_{\alpha } \sum\limits_{n = 1}^{N} {\alpha_{n} } - \frac{1}{2}\sum\limits_{n = 1}^{N} {\sum\limits_{m = 1}^{N} {\alpha_{m} \alpha_{n} y_{m} y_{n} x_{m}^{T} x_{n} } } \hfill \\ {\text{s}} . {\text{t}} .\left\{ {\begin{array}{*{20}c} {0 \le \alpha_{n} \le C} \\ {\sum\limits_{n = 1}^{N} {\alpha_{n} y_{n} } = 0} \\ \end{array} , \,\, n = 1, \ldots ,N} \right. \hfill \\ \end{array}$$

The key advantage of the dual form function is that the slack variables *ξ*_*n*_ vanish from the dual problem, with the constant *C* appearing only as an additional constraint on the Lagrange multipliers.

### Fuzzy SVM

Fuzzy SVM (FSVM) is more effective than standard SVM in predict or classify real-world data, in which a part of training points are less important than other points. We would like to force that the meaningful training points must be classified correctly and meaningless points like noises or outliers can be treated with less weight (Lin and Wang 2002).

FSVM applies a fuzzy membership function (FMF) *s* to each training data (Xian 2010), so that the training set is transformed into a fuzzy set, which can be expressed as

13 $$\left\{ {(x_{n} ,s_{n} ,y_{n} )|x_{n} \in {\mathbb{R}}^{p} ,\,0 < s_{n} \le 1,y_{n} \in \{ + 1, - 1\} } \right\},n = 1, \ldots ,N$$

where *s*_*n*_ is the altitude of the corresponding training point toward one class and (1 − *s*_*n*_) is the attitude of meaning less. The optimal hyperplane problem of FSVM is defined as:

14 $$\begin{array}{l} \mathop {\hbox{min} }\limits_{{{\mathbf{w}},\xi ,{\mathbf{b}}}} \frac{1}{2}\left\| {\mathbf{w}} \right\|^{2} + C{\mathbf{s}}^{T} {\varvec{\upxi}} \hfill \\ {\text{s}} . {\text{t}} . { }\left\{ {\begin{array}{*{20}c} {y_{n} \left( {{\mathbf{w}}^{T} x_{n} - {\mathbf{b}}} \right) \ge 1 - \xi_{n} } \\ {\xi_{n} \ge 0} \\ \end{array} } \right., \, n = 1, \ldots ,N \hfill \\ \end{array}$$

where **s** = (*s*_1_, *s*_2_, …, *s*_*N*_) represents the fuzzy membership vector. A smaller *s*_*n*_ reduces the effect of the parameter *ξ*_*n*_, such that the corresponding point *x*_*n*_ is treated less important. In a similar way, we construct the Lagrangian

15 $$\mathop {\hbox{min} }\limits_{{{\mathbf{w}},\xi ,{\mathbf{b}}}} \mathop {\hbox{max} }\limits_{\alpha ,\beta } \left\{ {\frac{1}{2}\left\| {\mathbf{w}} \right\|^{2} + C{\mathbf{s}}^{T} {\varvec{\upxi}} - \sum\limits_{n = 1}^{N} {\beta_{n} \xi_{n} } - \sum\limits_{n = 1}^{N} {\alpha_{n} \left[ {y_{n} \left( {{\mathbf{w}}^{T} x_{n} - {\mathbf{b}}} \right) - 1 + \xi_{n} } \right]} } \right\}$$

Again, the dual form is used to transform problem (15) to

16 $$\begin{aligned} \mathop {\hbox{max} }\limits_{\alpha } \sum\limits_{n = 1}^{N} {\alpha_{n} } - \frac{1}{2}\sum\limits_{n = 1}^{N} {\sum\limits_{m = 1}^{N} {\alpha_{m} \alpha_{n} y_{m} y_{n} x_{m}^{T} x_{n} } } \hfill \\ s.t.\left\{ {\begin{array}{*{20}c} {0 \le \alpha_{n} \le s_{n} C} \\ {\sum\limits_{n = 1}^{N} {\alpha_{n} y_{n} } = 0} \\ \end{array} , \,\, n = 1, \ldots ,N} \right. \hfill \\ \end{aligned}$$

### Fuzzy membership

We set the FMF as a distance function between the point and its class center. Suppose the mean of class +1 as *x*_+_ and the mean of class −1 as *x*_−_. Then we can get the radius of two classes as

17 $$r_{ - } = \mathop {\hbox{max} }\limits_{{\{ x_{n} :y = - 1\} }} \left| {x_{ - } - x_{n} } \right|$$

18 $$r_{ + } = \mathop {\hbox{max} }\limits_{{\{ x_{n} :y = + 1\} }} \left| {x_{ + } - x_{n} } \right|$$

The fuzzy membership *s*_*n*_ is defined as a function of the radius and mean of each class (Lin and Wang 2002)

19 $$s_{n} = \left\{ {\begin{array}{*{20}c} {1 - \left| {x_{ + } - x_{n} } \right|/(r_{ + } + \delta )} & {y_{n} = + 1} \\ {1 - \left| {x_{ - } - x_{n} } \right|/(r_{ - } + \delta )} & {y_{n} = - 1} \\ \end{array} } \right.$$

where *δ* > 0 is used to guarantee *s*_*n*_ > 0.

## Implementation and experiments

### Implementation

**Fig. 3:**
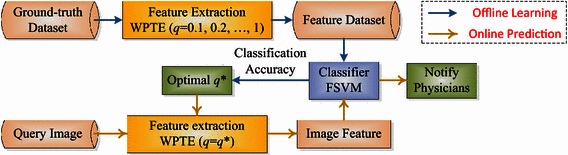
Diagram of the proposed PBD system

Figure 3 shows the diagram of the proposed PBD system. In the offline learning phase, the users expect to select the optimal *q* (to determine the value of q*), and train the classifier. In the online prediction phase, the users will get the prediction results for each query image.

### Experiment design

In this study, we developed four different methods. “WPSE + SVM”, “WPSE + FSVM”, “WPTE + SVM”, and “WPTE + FSVM”. Theoretically, the last one will perform the best since WPSE in a special case of WPTE, and FSVM is an extension of SVM with additional ability to reduce influences from noises and outliers.

We need to prove it by experiments. In this work, we designed five tasks. (1) We gave a comparison between DWT and WPT. A healthy brain and a pathological brain were used. We use a 2-level Haar wavelet decomposition. (2) We compared the proposed WPSE and WPTE features with traditional DWT and “DWT + PCA”. All used SVM as classifiers (3) We compared the four proposed classifiers, to check whether FSVM is superior to SVM. (4) We selected the best of proposed methods, and compared it with state-of-the-art approaches. (5) We used grid searching to find the optimal parameter of *q*.

## Results and discussions

The experiments were carried out on the platform of IBM machine with 3 GHz core i3 processor and 8 GB random access memory (RAM), running under Windows 7 operating system (OS). The algorithm was developed by ourselves based on the platform of Matlab 2014a (The Mathworks ©).

### WPT versus DWT

**Fig. 4:**
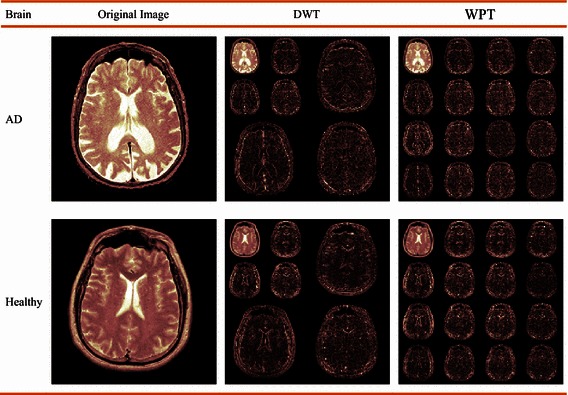
Decompositions comparison between DWT and WPT

In the first experiment, we compared DWT with WPT on a healthy brain and an Alzheimer’s disease brain, respectively. The second column shows the original image, the third column the DWT decomposition results, and the final column the WPT results. Pink colormap is employed for better view (Fig. 4).

### Feature comparison

In the second experiment, we compared the proposed WPSE and WPTE (*q* is set to 0.8, please refer to "Optimal parameter q"), with two types of traditional features: (i) DWT and (ii) DWT + PCA. (Note that Chaplot et al. (2006) proposed the DWT + SVM method, Zhang and Wu (2012) proposed DWT + PCA + SVM method). For fair comparison, we choose the same classifier—SVM.

Table 4 shows that both “WPSE + SVM” achieves accuracies of 98.64, 97.12, and 97.02 % on D-66, D-160, and D-255, respectively. The “WPTE + SVM” achieves accuracies of 99.09, 98.94, and 98.39 % over three datasets. The results are better than those obtained either by “DWT + SVM (Chaplot et al. 2006)” or by “DWT + PCA + SVM (Zhang and Wu 2012)”. Therefore, we can conclude that WPSE and WPTE excel traditional feature extraction methods of “DWT” and “DWT + PCA”. Particularly, WPTE is better than WPSE. The reason is three-fold: (1) TE is a generalization of traditional SE (Tsallis 2014), and TE had been successfully applied in brain images (Amaral-Silva et al. 2014; Venkatesan and Parthiban 2014; Khader and Ben Hamza 2011). (2) The combination of TE and wavelet transform had proven to perform better than either TE or DWT in other applications (Hussain 2014; Liu et al. 2014; Chen and Li 2014). (3) Brain images entail long-range interaction and fractal-type structure, because of the self-similarity observed brain structures imaged with a finite resolution, which can be easily extracted by the corresponding wavelet packet coefficients. In summary, there are similarities at different spatial scales in brain images, which makes WPTE more suitable than WPSE in describing brains.

**Table 4:** Feature comparison with SVM as classifier (K-fold SCV)

Approaches	Feature #	Run #	D-66	D-160	D-255
DWT+SVM (Chaplot et al. 2006)	4761	5	96.15	95.38	94.05
DWT + PCA + SVM (Zhang and Wu 2012)	19	5	96.01	95.00	94.29
WPSE + SVM (proposed)	16	10	98.64	97.12	97.02
WPTE + SVM (proposed)	16	10	99.09	98.94	98.39

### Classifier comparison

To compare the classification performance between SVM and FSVM. We set the features as WPSE and WPTE (*q* = 0.8). Then, we applied both SVM and FSVM for classification. The 10 runs of K-fold SCV results are listed below in Table 5.

**Table 5:** SVM versus FSVM (10xK-fold SCV)

Proposed approaches	D-66	D-160	D-255
WPSE + SVM	98.64	97.12	97.02
WPSE + FSVM	99.85	99.69	98.94
WPTE + SVM	99.09	98.94	98.39
WPTE + FSVM	100.00	100.00	99.49

Results in Table 5 shows that “WPSE + FSVM” obtains accuracies of 99.85, 99.69, 98.94 % over three datasets, which are higher than those obtained by “WPSE + SVM”. The similar results occur between “WPTE + FSVM” and “WPTE + SVM” in the way that the classification accuracy increases after SVM is replaced with FSVM. The reason is FSVM applies a FMF to each training data, so FSVM can reduce the influence of noises and outliers. In addition, the “WPTE + FSVM” performs the best among all four proposed approaches. It will be used as the default proposed method in following text.

### Comparison with state-of-the-art

We compared the best proposed method (WPTE + FSVM), with 17 recent proposed methods, which consist of DWT + SOM (Chaplot et al. 2006), DWT + SVM (Chaplot et al. 2006), DWT + SVM + RBF (Chaplot et al. 2006), DWT + SVM + POLY (Chaplot et al. 2006), DWT + PCA + KNN (El-Dahshan et al. 2010), DWT + PCA + FP-ANN (El-Dahshan et al. 2010), DWT + PCA + SCG-FNN (Dong et al. 2011), DWT + PCA + SVM (Zhang and Wu 2012), DWT + PCA + SVM + RBF (Zhang and Wu 2012), DWT + PCA + SVM + IPOL (Zhang and Wu 2012), DWT + PCA + SVM + HPOL (Zhang and Wu 2012), RT + PCA + LS-SVM (Das et al. 2013), DWT + SE + SWP + PNN (Saritha et al. 2013), PCNN + DWT + PCA + BPNN (El-Dahshan et al. 2014), SWT + PCA + IABAP-FNN (Wang et al. 2015a), SWT + PCA + ABC-SPSO-FNN (Wang et al. 2015a), and WE + HMI + GEPSVM (Zhang et al. 2015d).

We averaged the results of 10 runs of K-fold SCV. The comparison results are listed in Table 6, in which some old approaches ran five times in their papers with results extracted from literature (Das et al. 2013). This experiment ran ten times to get more robust results than a five-time run.

**Table 6:** Classification comparison

Existing approaches	Feature #	Run #	D-66	D-160	D-255
DWT + SOM (Chaplot et al. 2006)	4761	5	94.00	93.17	91.65
DWT + SVM (Chaplot et al. 2006)	4761	5	96.15	95.38	94.05
DWT + SVM + RBF (Chaplot et al. 2006)	4761	5	98.00	97.33	96.18
DWT + SVM + POLY (Chaplot et al. 2006)	4761	5	98.00	97.15	96.37
DWT + PCA + KNN (El-Dahshan et al. 2010)	7	5	98.00	97.54	96.79
DWT + PCA + FP-ANN (El-Dahshan et al. 2010)	7	5	97.00	96.98	95.29
DWT + PCA + SCG-FNN (Dong et al. 2011)	19	5	*100.00*	99.27	98.82
DWT + PCA + SVM (Zhang and Wu 2012)	19	5	96.01	95.00	94.29
DWT + PCA + SVM + RBF (Zhang and Wu 2012)	19	5	*100.00*	99.38	98.82
DWT + PCA + SVM + IPOL (Zhang and Wu 2012)	19	5	*100.00*	98.12	97.73
DWT + PCA + SVM + HPOL (Zhang and Wu 2012)	19	5	98.34	96.88	95.61
RT + PCA + LS-SVM (Das et al. 2013)	9	5	*100.00*	*100.00*	99.39
DWT + SE + SWP + PNN (Saritha et al. 2013)	3	5	*100.00*	99.88	98.90
PCNN + DWT + PCA + BPNN (El-Dahshan et al. 2014)	7	10	*100.00*	98.88	98.24
SWT + PCA + IABAP-FNN (Wang et al. 2015a)	7	10	*100.00*	99.44	99.18
SWT + PCA + ABC-SPSO-FNN (Wang et al. 2015a)	7	10	*100.00*	99.75	99.02
WE + HMI + GEPSVM (Zhang et al. 2015d)	14	10	*100.00*	99.56	98.63

Proposed approach	Feature #	Run #	D-66	D-160	D-255
WPTE + FSVM	16	10	*100.00*	*100.00*	*99.49*

The italic represents the highest accuracy among all algorithms

The value of *q* was again assigned with 0.8 (The reason can be found in “Optimal parameter q”). The regularization constant *C* were obtained via grid-search method.

Table 6 shows the proposed “WPTE + FSVM” performed better than existing state-of-the-art methods, obtaining perfect classification for the first two datasets and an accuracy of 99.49 % for D-255. This demonstrated the effectiveness of FSVM, which can reduce the effect of noise and outliers in the training points, yielding a more reliable hyperplane than standard SVM. The second best classifier is “RT + PCA + LS-SVM” (Das et al. 2013) that achieved 99.39 % for D-255.

Finally, the average evaluations based on 10 runs of the proposed WPTE + FSVM method were listed in Table 7. For D-66 and D-160, the WPTE + FSVM yielded perfect classification. For the D-255, its performance slightly decreased with sensitivity of 99.50 %, specificity of 99.43 %, precision of 99.91 %, and accuracy of 99.49 %.

**Table 7:** Average evaluation of WPTE + FSVM method based on 10 runs

	Sensitivity	Specificity	Accuracy	Precision
D-66	Perfect			
D-160	Perfect			
D-255	99.50	99.43	99.49	99.91

### Optimal parameter *q*

**Fig. 5:**
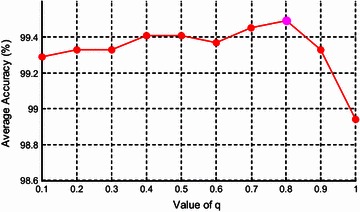
Effect of *q* on average accuracy

The parameter *q* influences the extracted features, so it also influences classification performance. Its value should be no more than 1, since the brain image is subextensive, containing complicated regions. In this final experiment, we varied the value of *q* in the set of [0.1, 0.2, 0.3, …, 0.1, 1] (Note *q* = 1 degrades WPTE to WPSE), and ran the offline training for each value. We recorded the average accuracy over 10 runs on the dataset D-255 by the proposed “WPTE + FSVM”. The results are shown in Fig. 5 and Table 8.

**Table 8:** The average accuracy changes with the value of *q*

*q*	Average accuracy
0.1	99.29
0.2	99.33
0.3	99.33
0.4	99.41
0.5	99.41
0.6	99.37
0.7	99.45
0.8	99.49
0.9	99.33
1.0	98.94

Figure 5 demonstrates the value of *q* yields slight but discernible effect on average accuracy of 10 runs. As *q* increases to 0.8, the curve increases gradually till the highest. As *q* increases to 0.1, the average accuracy decreases sharply. The result again validates that WPTE (*q* = 0.8) is better than WPSE (*q* = 1).

This optimal result (*q* = 0.8) in this work exactly identical to three recent literatures: Sturzbecher et al. (2009), Cabella et al. (2009), and Zhang et al. (2015b). Furthermore, Diniz et al. (2010) found the fact that *q* = 1.5 for gray matter (GM), 0.1 for white matter (WM), and 0.2 for cerebrospinal fluid (CSF). Here we treat the whole brain as a single, so we must assign a single value to *q*. The optimal *q* of 0.8 can be regarded as an average of best *q* of GM, WM, and CSF.

### Discussion on the proposed method

There were three causes to use WPT, TE, and FSVM. (1) WPT yields more features than DWT does. (2) Entropy can efficiently represent the complexity of subband coefficients, and TE is a better feature descriptor for brain structures than SE. (3) FSVM applies a FMF to each training data, so it can reduce the influence of noises and outliers.

The contributions of this work centered in three points: (i) We employed WPTE that offered better information description than WPSE. (ii) We employed FSVM that can deal with noises and outliers compared to plain SVM; and (iv) We proved the proposed “WPTE + FSVM” approach obtained superior average accuracy to 17 state-of-the-art approaches.

## Conclusion and future research

In this study, we treated the PBD as a binary classification problem as pathological and healthy. To solve it, we proposed a novel feature WPTE, which used WPT to replace traditional DWT method and used TE to replace traditional SE method, and fed WPTE into FSVM. The experiments showed the proposed “WPTE + FSVM” method yielded superior performance to state-of-the-art methods.

Future work should focus on the following four aspects: (i) we will include other imaging techniques, such as DTI, FMRI and MRSI; (ii) the classification performance may increase by using other advanced variants of SVMs, such as GEPSVM (Yu et al. 2015a) and Twin SVM (Jayadeva et al. 2007). (iii) we will check the effect produced by other wavelet family and other decomposition levels. (iv) We will try to develop fine-grid search to replace the coarse-grid search technique. (v) Swarm intelligence methods (Wang et al. 2015b) will be employed to train the weights of classifiers.

## Abbreviations

(A)(BP)(F)NN: (artificial) (back-propagation) (feed-forward) neural network
(D)W(P)T: (discrete) wavelet (packet) transform
(k)(F)SVM: (kernel) (Fuzzy) support vector machine
(G)(W)M: (gray) (white) matter
(S)(T)E: (shannon) (tsallis) entropy
ABC: artificial bee colony
AD: Alzheimer’s disease
BBO: biogeography-based optimization
CAD: computer-aided diagnosis
CSF: cerebrospinal fluid
FMF: fuzzy membership function
HD: huntington’s disease
KNN: K-nearest neighbor
MR(I): magnetic resonance (imaging)
MS: multiple sclerosis
PiD: pick’s disease
PBD: pathological brain detection
PNN: probabilistic neural network
QMF: quadrature mirror filter
RT: ripplet transform
SCV: stratified cross validation
SDH: subdural hematoma
SOM: self-organizing map
WP(S)(T)E: wavelet packet (Shannon) (Tsallis) entropy
